# Hearing loss and cognitive decline: why Brazil needs a national campaign

**DOI:** 10.1016/j.bjorl.2026.101816

**Published:** 2026-04-13

**Authors:** Rogério Hamerschmidt, Vagner Antonio Rodrigues Silva, Arthur Menino Castilho

**Affiliations:** a Universidade Federal do Paraná (UFPR), Department of Otolaryngology, Curitiba, PR, Brazil; b Universidade de Campinas (Unicamp), Faculdade de Ciências Médicas (FCM), Department of Otolaryngology, Head and Neck Surgery, Campinas, SP, Brazil

Hearing loss (HL) affects approximately 20% of the global population, exceeding 1.5 billion individuals and continuing to rise, largely driven by population aging. By 2050, an estimated 2.45 billion people will be living with hearing loss, nearly 700 million of whom will have moderate to profound impairment.^1^ HL is the leading modifiable risk factor for dementia, accounting for approximately 8% of potentially preventable cases. The mechanisms underlying this association are well established: increased cognitive load due to impaired auditory processing, sensory deprivation leading to brain atrophy, and social isolation resulting from communication difficulties all contribute to cognitive decline.^2^

Dementia represents one of the greatest public health challenges worldwide.^3^ In 2021, 57 million people were living with dementia, with projections suggesting this number may nearly triple by 2050. It is a leading cause of mortality and disability globally.^4^ Meta-analyses have demonstrated a significant increase in the risk of dementia (HR 1.35), mild cognitive impairment (HR 1.29), and Alzheimer’s disease (HR 1.56) among individuals with hearing loss.^5^ A clear dose–response relationship has been observed, with a 16% increase in dementia risk for every 10 dB worsening in hearing thresholds.

Epidemiological data in Brazil remain limited and are largely based on self-reported measures, which likely underestimate the true prevalence. Population-based studies suggest a self-reported prevalence of hearing loss of approximately 5.2%^6^ in the general population and 30.4% among older adults — figures that are likely underestimated when compared with global data. Structural factors further exacerbate the Brazilian scenario, including unequal access to hearing healthcare services, limited coverage of early diagnosis, restricted availability of rehabilitation, and the absence of systematic population-based screening strategies. The rapid aging of the Brazilian population is expected to further increase the burden of hearing loss in the coming decades.

The ACHIEVE study^7^ demonstrated that hearing interventions, including hearing aid use combined with auditory rehabilitation, can reduce cognitive decline over a three-year period, particularly among high-risk individuals. However, adherence to treatment remains extremely low, with only 10–30% of eligible individuals using hearing aids, even in high-income countries. HL can no longer be regarded as an isolated condition or a natural process that does not require treatment. It is a condition with direct impact on cognitive health, functional status, and quality of life. More importantly, it represents a tangible opportunity for intervention targeting one of the main determinants of dementia.

Brazil has a strong track record of successful national public health campaigns, including those focused on breast cancer, HIV/AIDS, and vaccination. These initiatives have demonstrated that coordinated, evidence-based strategies with broad public engagement can effectively modify behavior, expand diagnosis, and reduce disease burden at a population level. A national campaign addressing HL and cognition is therefore warranted. Its main objectives should include: (1) increasing public awareness of the association between hearing loss and cognitive decline; (2) promoting early diagnosis, particularly within primary care; (3) expanding access to hearing rehabilitation, including hearing aids and implantable technologies; (4) reducing the stigma associated with hearing device use; and (5) integrating hearing health into broader aging and cognitive health policies, recognizing their interdependence.

The integration of otorhinolaryngology, geriatrics, neurology, and public health will be essential for the success of such an initiative. The involvement of medical societies, healthcare managers, and policymakers is also crucial. Ignoring hearing loss as a modifiable risk factor for dementia represents a missed opportunity for large-scale intervention. Recognizing its role and acting in a structured manner may significantly contribute to reducing the burden of dementia in Brazil in the context of an aging population. Hearing better may ultimately mean living better—and thinking longer.

## ORCID ID

Rogério Hamerschmidt: 0000-0002-7722-6409

Vagner Antonio Rodrigues Silva: 0000-0002-7335-4489

Arthur Menino Castilho: 0000-0002-9024-8004

## Funding

The authors have no financial relationships relevant to this article to disclose.

## Data availability statement

N/A.

## Declaration of competing interest

The authors declare no conflicts of interest.
